# Serum Starvation Enhances the Antitumor Activity of Natural Matrices: Insights into Bioactive Molecules from Dromedary Urine Extracts

**DOI:** 10.3390/molecules30040821

**Published:** 2025-02-10

**Authors:** Maria Noemi Sgobba, Biagia Musio, Carlos Iglesias Pastrana, Stefano Todisco, Nikola Schlosserovà, Federica Mastropirro, Maria Favia, Antonio Radesco, Iola F. Duarte, Anna De Grassi, Mariateresa Volpicella, Vito Gallo, Ciro Leonardo Pierri, Elena Ciani, Lorenzo Guerra

**Affiliations:** 1 Department of Biosciences, Biotechnologies and Environment, University of Bari “Aldo Moro”, Via Orabona 4, 70125 Bari, Italyf.mastropirro@phd.uniba.it (F.M.); anna.degrassi@uniba.it (A.D.G.); mariateresa.volpicella@uniba.it (M.V.); elena.ciani@uniba.it (E.C.); lorenzo.guerra1@uniba.it (L.G.); 2 Department of Civil, Environmental, Land, Building Engineering and Chemistry (DICATECh), Polytechnic University of Bari, Via Orabona 4, 70125 Bari, Italy; biagia.musio@poliba.it (B.M.); stefano.todisco@poliba.it (S.T.); vito.gallo@poliba.it (V.G.); 3 Faculty of Veterinary Sciences, Department of Genetics, University of Córdoba, 14071 Córdoba, Spain; ciglesiaspastrana@gmail.com; 4 Department of Translational Biomedicine and Neurosciences (DiBraiN), University of Bari “Aldo Moro”, Piazza Giulio Cesare, 70124 Bari, Italy; maria.favia@uniba.it; 5 Istituto Tumori “Giovanni Paolo II” I.R.C.C.S., Viale Orazio Flacco 65, 70124 Bari, Italy; 6 Department of Chemistry, CICECO—Aveiro Institute of Materials and LAQV-REQUIMTE, University of Aveiro, 3810-193 Aveiro, Portugal; ioladuarte@ua.pt; 7 Innovative Solutions S.r.l.—Spin-Off Company of the Polytechnic University of Bari, Zona H 150/B, 70015 Noci, Italy; 8 Department of Pharmacy—Pharmaceutical Sciences, University of Bari Aldo Moro, Via Orabona 4, 70125 Bari, Italy

**Keywords:** natural matrices, dromedary urine extracts, serum-reduced conditions, human renal cells, cell viability, apoptosis, NMR metabolomics, azelaic acid

## Abstract

Natural matrices have historically been a cornerstone in drug discovery, offering a rich source of structurally diverse and biologically active compounds. However, research on natural products often faces significant challenges due to the complexity of natural matrices, such as urine, and the limitations of bioactivity assessment assays. To ensure reliable insights, it is crucial to optimize experimental conditions to reveal the bioactive potential of samples, thereby improving the validity of statistical analyses. Approaches in metabolomics further strengthen this process by identifying and focusing on the most promising compounds within natural matrices, enhancing the precision of bioactive metabolite prioritization. In this study, we assessed the bioactivity of 17 dromedary urine samples on human renal cells under serum-reduced conditions (1%FBS) in order to minimize possible FBS-derived interfering factors. Using viability assays and Annexin V/PI staining, we found that the tumor renal cell lines Caki-1 and RCC-Shaw were more sensitive to the cytotoxic effects of the small molecules present in dromedary urine compared to non-tumor HK-2 cells. Employing NMR metabolomics analysis combined with detected in vitro activity, our statistical model highlights the presence of bioactive compounds in dromedary urine, such as azelaic acid and phenylacetyl glycine, underscoring its potential as a sustainable source of bioactive molecules within the framework of green chemistry and circular economy initiatives.

## 1. Introduction

Natural matrices have long been esteemed as a source of metabolites, providing a wide range of bioactive compounds with potential therapeutic benefits [1,2]. In this context, the urine of various animals, such as goats, sheep, buffalo, elephants, horses, camels, and donkeys, have historically been utilized as a natural remedy for numerous health issues, including abdominal tumors, tuberculosis, anemia, and infectious diseases [3,4,5]. In particular, dromedary urine has been traditionally used in various Arabian countries for its medicinal properties and is now gaining scientific attention for its unique metabolite profile [3,5]. Dromedary urine is considered the best candidate for urotherapy, as it appears to possess more beneficial effects than other animal urine [6,7]. Indeed, researchers have demonstrated that dromedary urine can exhibit antimicrobial, antibacterial, antifungal, anticancer, and antiviral properties, making it a subject of interest in natural product research [3,4,5,8,9,10]. However, the efficacy and reproducibility of these studies can vary significantly depending on the animal species and the environment in which they live; thus, the metabolome of a biological system is defined by a set of end products deriving from physiological and environmental stimuli [11]. This variability underscores the importance of considering both biological and environmental factors affecting the therapeutic potential of natural products [7,12,13]. In this regard, metabolomics offers the opportunity to gain insights into metabolites that can vary based on a variety of factors, such as diet, intestinal microbiota, physical activity, and environmental exposures. Metabolomics analysis can specifically contribute to the decomposition of natural matrices’ complexity and can facilitate the identification of bioactive compounds within dromedary urine by matching the information of dromedary urine biological activities with their chemical composition [14].

Additionally, in vitro studies are affected by a further source of variability [15], such as fetal bovine serum (FBS), a widely used cell culture medium supplement that supports cell growth. The serum is a critical factor that significantly influences bioactivity assay outcomes, especially when dealing with complex natural matrices like dromedary urine. Indeed, FBS is known to contain a wide range of growth factors, hormones, and other macromolecules that support cell growth and maintenance. However, its variability and potential to influence experimental outcomes necessitate the careful optimization of its concentration in culture media [16,17,18]. Studies have shown that the presence of FBS can impact the pharmacodynamics of tested compounds, potentially masking or enhancing their bioactivity and affecting their availability and interaction with target cells [19,20]. Moreover, FBS can influence cellular responses, such as proliferation, differentiation, and metabolic activity, further complicating the interpretation of results [17]. Furthermore, the natural matrix under investigation necessitates careful consideration of its unique physico-chemical properties, such as osmolarity [3,10], thus highlighting the importance of optimizing experimental conditions to accurately assess the bioactive potential of these animal-derived metabolites.

In this work, we tested the bioactive potential of a set of 17 urine samples from dromedaries of different sex, age, and physiological status on three human renal cell lines, the non-tumor HK2 and the tumor Caki-1 and RCC-Shaw cell lines, by carefully considering the possible influence of FBS concentration in the in vitro bioassays, thus performing all the assays under conditions of serum starvation (1% FBS). Moreover, in order to explore the presence of potential bioactive compounds, the chemical composition of dromedary urine was deeply investigated using a non-targeted Nuclear Magnetic Resonance (NMR)-based approach.

## 2. Results

### 2.1. Impact of FBS Concentration on Renal Cell Viability

**Figure 1:**
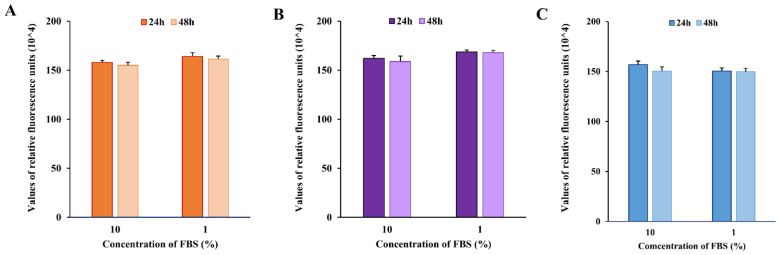
Alamar Blue assay after exposure to serum-reduced cell culture medium. Human renal cells (**A**) HK-2, (**B**) Caki-1, (**C**) RCC-Shaw were exposed to a 10% FBS-supplemented cell culture medium (control) and to a 1% FBS-supplemented medium (serum-reduced condition) for 24 and 48 h. No significant impact on renal cell viability was observed in serum-reduced conditions compared to the control. Cell viability was measured using the values of relative fluorescence units (RFU). The statistical significance of the RFU of the treated cells compared to the control ones was assessed using the Mann–Whitney test (*n* for each condition is included within 25 and 33 observations from at least 3 independent experiments).

The use of serum, especially fetal bovine serum (FBS), is essential to in vitro animal cell cultures, though no standard method exists to assess the potential adverse effects of unknown factors present in the complex composition of FBS in the experiments [16]. Bearing in mind the issues related to the employment of FBS, we first assessed whether reducing the FBS concentration from 10% to 1% could affect HK-2, Caki-1, and RCC-Shaw cell viability. The Alamar Blue assay was performed after 24 and 48 h of exposure of cells to 1% FBS-supplemented cell culture medium, and the results showed, in all the three considered cell lines, no significant alterations in cells’ metabolic activity or viability, compared to the control condition, which is represented by the standard FBS concentration of 10% *v/v* (Figure 1). Considering the minimal impact on cell viability observed with 1% FBS, all experiments were conducted under serum-reduced conditions to explore the potential bioactivity of dromedary urine on human renal cells in a low-interference experimental condition. The Alamar Blue assay highlights no significant alteration in cell viability in either non-tumor HK-2 cells or the tumor Caki-1 and tumor RCC-Shaw cell lines.

### 2.2. Impact of Hyperosmotic Stress on Renal Cell Viability in Low-Serum Conditions

Considering the strong water reabsorption capacity of dromedary kidneys [21] and the highly concentrated urine that this species can excrete [13], the osmolarity of the tested dromedary urine samples was measured before cell exposure. We found great variability in the urine concentration, with values between 424 and 1976 mOsm/L, as reported in Table 1.

The in vitro exposure of cells to hyperosmotic stress induces a cascade of cellular responses. These include cell shrinkage, oxidative stress, protein carbonylation, mitochondrial depolarization, DNA damage, and cell cycle arrest, consequently impeding cell proliferation and enhancing cell susceptibility to apoptosis [22,23,24,25]. In order to exclude the possible impact of urine hyperosmolarity on the viability of the investigated cell lines, a series of cell viability experiments were carried out to identify the tolerance threshold of the non-tumor renal cell line HK-2 and the tumor renal cells Caki-1 and RCC-Shaw when exposed to solutions with osmolarities exceeding the physiological value of about 290 mOsm/L in serum-reduced conditions. To simulate hyperosmolarity, D-(-)-mannitol was used as an inert, and cell-impermeable osmolyte [26] was dissolved at various concentrations in the 1% FBS-supplemented cell culture medium. In this way, the complete cell culture medium osmolarity was increased from the physiological value of 290 mOsm/L up to 400, 450, 500, 600, and 700 mOsm/L.

**Figure 2:**
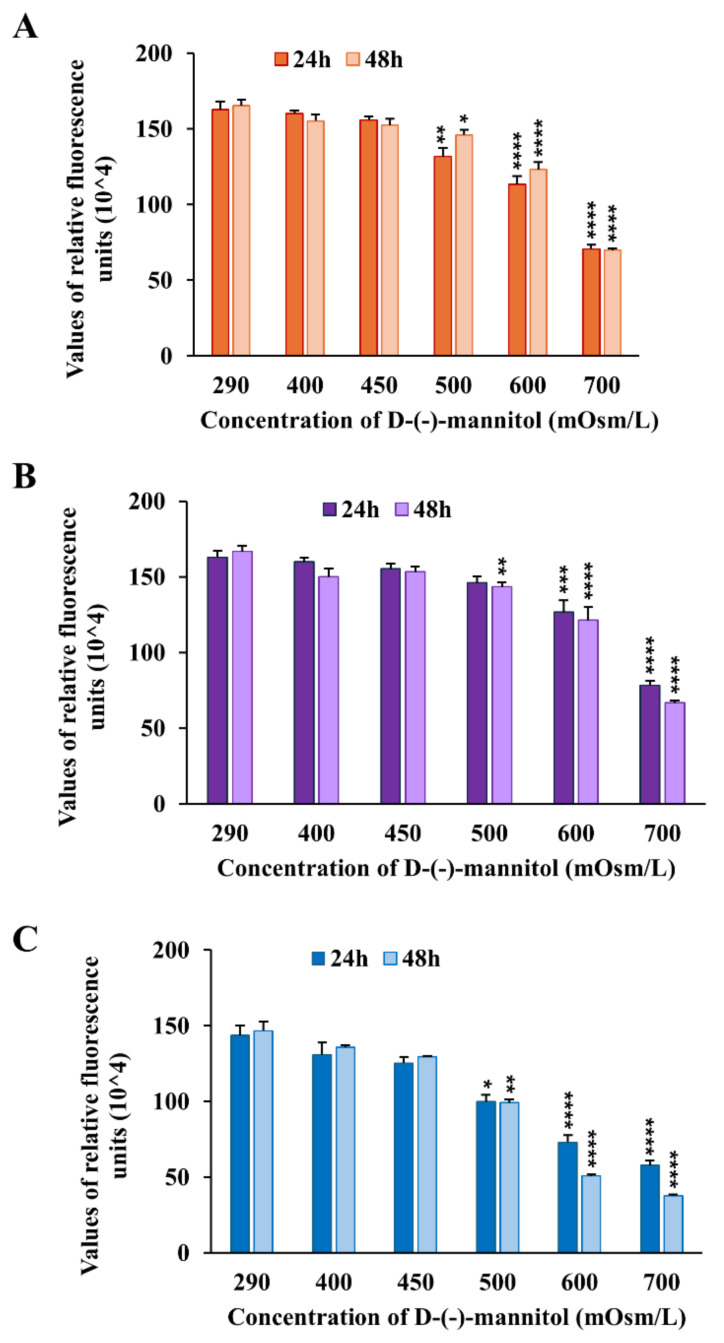
Alamar Blue assay after treatment with a D-(-)-mannitol-composed medium. Human renal cells (**A**) HK-2, (**B**) Caki-1, and (**C**) RCC-Shaw were exposed to 1% FBS-supplemented hyperosmolar solutions for 24 and 48 h. All three considered cell lines exhibited no significant decline in cell viability decline, compared to the control condition, up to 450 mOsm/L, thus identifying this value as the hyperosmolarity tolerance threshold. Cell viability was measured using the values of relative fluorescence units (RFU). The statistical significance of the RFU of the treated cells compared to the control ones was assessed through the Mann–Whitney test (*n* for each condition is included within 9 and 15 observations, from at least 3 independent experiments; * *p* < 0.05, ** *p* < 0.01, *** *p* < 0.001, **** *p* < 0.0001).

HK-2, Caki-1, and RCC-Shaw cells were seeded in 96 multi-well plates to undergo the Alamar Blue cell viability assay after 24 and 48 h of exposure to D-(-)-mannitol-composed hyperosmotic solutions and to the control condition, represented by the 1% FBS cell culture medium. A statistically significant reduction in cell viability was observed, compared to the control condition, in all three renal cell lines for the 500, 600, and 700 mOsm/L solutions after both 24 and 48 h of treatment (Figure 2). Notably, after 48 h of incubation at the highest concentration (700 mOsm/L), cell viability was approximately 40% in HK2 and Caki-1 cells and 23% in RCC cells (Figure 2). The observed results suggest a value of 450 mOsm/L as the hyperosmolarity tolerance threshold for the investigated cellular models. Therefore, hyperosmolar dromedary urine samples (as reported in Table 1) were opportunely diluted to make them remain under this threshold.

### 2.3. Impact of Dromedary Urine Solutions on Renal Cell Viability

**Figure 3:**
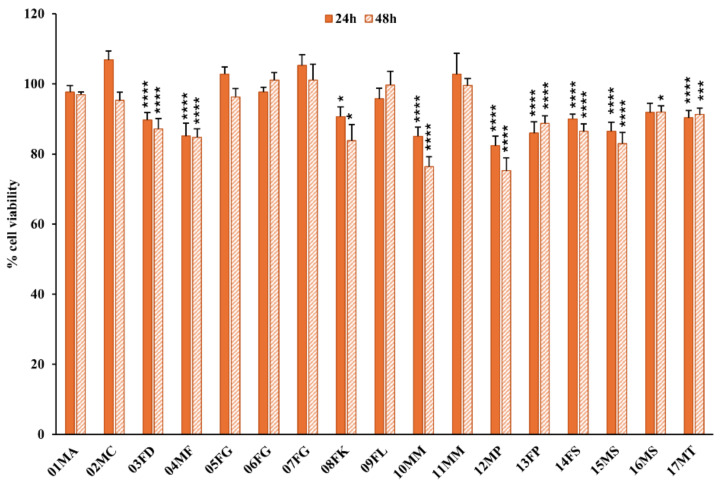
Cell viability assay on non-tumor HK-2 cells after treatment with dromedary urine samples. The non-tumor cell line after 24 and 48 h of exposure to dromedary urine exhibited a statistically significant viable decline in 8 out of the 17 tested samples. Data are reported as the percentage of the mean and the standard error of the mean (SEM) compared to the respective control conditions (non-exposed cells), and samples were labeled as (i) inactive when *p* > 0.01, (ii) mildly bioactive when 0.01 < *p* < 0.0001, and (iii) bioactive when *p* < 0.0001 (Mann–Whitney test, XLSTAT, *n* = 9–15; * *p* < 0.05, *** *p* < 0.001, **** *p* < 0.0001).

Based on the aforementioned results, all dromedary urine samples were diluted in a 1% FBS-supplemented cell culture medium to reduce the osmolarity values below the identified tolerance threshold of 450 mOsm/L. Hence, the cell lines HK-2, Caki-1, and RCC-Shaw were seeded in 96 multi-well plates for exposure to dromedary urine solutions for 24 and 48 h, and the Alamar Blue assay was performed at each time point. The results from the viability assay performed on the non-tumor HK-2 cells, as summarized in Figure 3 (and detailed in Table S1), indicated that 8 of the 17 tested urine samples exhibited a statistically significant reduction in cell viability at 24 and 48 h post-treatment. Some of the bioactive urine samples, i.e., 03FD, 14FS, and 17MT, induced a viable decline of roughly 10%, while the strongest impact on HK-2 was observed for the sample 12MP, which reduced the non-tumor cell viability by up to 25% at 48 h (Figure 3, Table S1).

**Figure 4:**
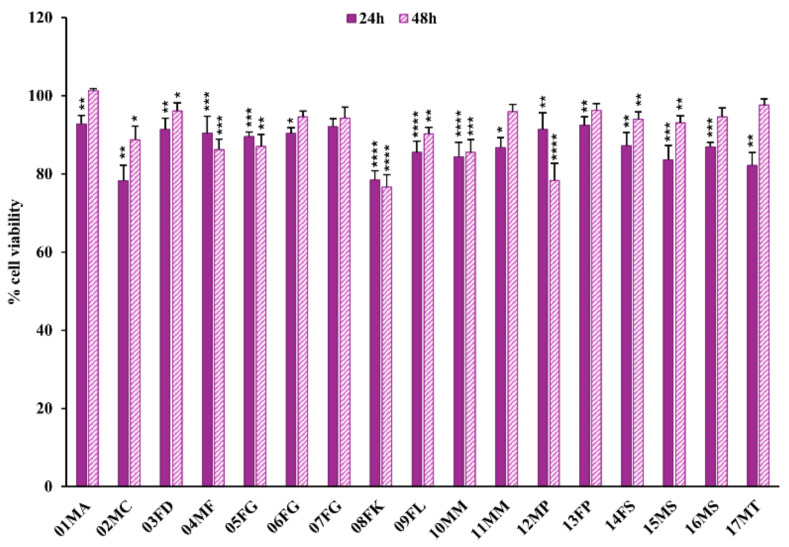
**Cell viability assay on tumor Caki-1 cells after treatment with dromedary urine samples**. The tumor renal cells Caki-1 showed a weak but statistically significant decline in viability in almost all of the tested urine samples after 24 h of incubation. The observed cytotoxic effect remained only for 8 of the bioactive urine samples after 48 h of exposure. Data are reported as the percentage of the mean and the standard error of the mean (SEM) compared to the respective control conditions (non-exposed cells), and samples were labeled as (i) inactive when *p* > 0.01, (ii) mildly bioactive when 0.01 < *p* < 0.0001, and (iii) bioactive when *p* < 0.0001 (Mann–Whitney test, XLSTAT, *n* = 9–18; * *p* < 0.05, ** *p* < 0.01, *** *p* < 0.001, **** *p* < 0.0001).

In the case of Caki-1 tumor renal cells, the Alamar Blue assay revealed a statistically significant reduction in cell viability following 24 h of exposure to all the urine samples, except for 07FG and 11MM, as reported in Figure 4 (and detailed in Table S2). However, extending the incubation of Caki-1 cells up to 48 h with the same urine solutions, a significant decrease in cell viability persisted only for 8 of the 16 bioactive urine solutions, i.e., from 04MF, 05FG, 08FK, 09FL, 10MM, 12MP, 14FS, and 15MS dromedaries, while 01MA, 02MC, 03FD, 06FG, 13FP, 16MS, and 17MT lost their effect (Figure 4, Table S2).

**Figure 5:**
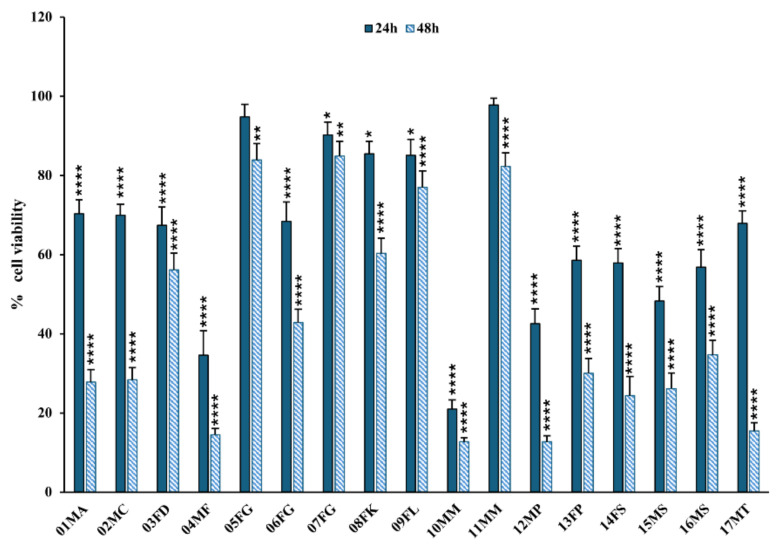
Cell viability assay on tumor RCC-Shaw cells after treatment with dromedary urine samples. Dromedary urine drastically impacted tumor renal cell viability; thus, all the tested samples induced a strong and statistically significant decrease in cell viability after 48 h of incubation. In particular, urine samples from 04MF, 10MM, 12MP, and 17MT demonstrated a residual cell viability of less than 20%. Data are reported as the percentage of the mean and the standard error of the mean (SEM) compared to the respective control conditions (non-exposed cells), and samples were labeled as (i) inactive when *p* > 0.01, (ii) mildly bioactive when 0.01 < *p* < 0.0001, and (iii) bioactive when *p* < 0.0001 (Mann–Whitney test, XLSTAT, *n* = 9–12; * *p* < 0.05, ** *p* < 0.01, **** *p* < 0.0001).

When the same experiments were performed on RCC-Shaw cells, a primary tumor-derived renal cell line, a remarkable reduction in viability was observed. In particular, after 24 h of incubation with 13 out of the 17 tested dromedary urine solutions, a significant cytotoxic effect was detected, with a decrease in cell viability of up to 79%, as observed for the urine solution from the 10MM dromedary compared to the control condition (Figure 5, Table S3). At 48 h of incubation with the same urine solutions, an even greater decline in viability was observed compared to the first time point. All the tested urine solutions exhibited a strong and statistically significant cytotoxic effect, with noticeably low residual viability, i.e., 14.5%, 12.6%, 12.7%, and 15.5% for 04MF, 10MM, 12MP, and 17MT, respectively, as shown in Figure 5 (and detailed in Table S3).

**Figure 6:**
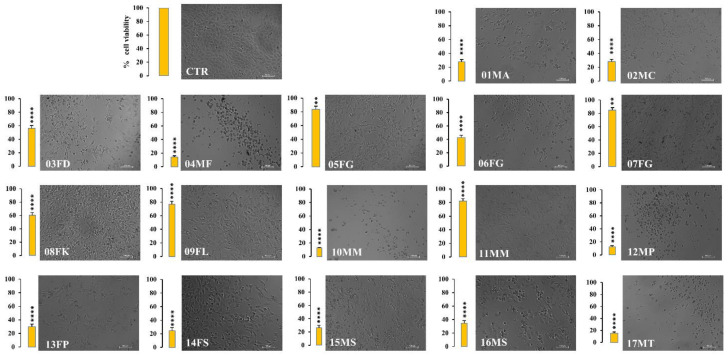
Optical microscopy analysis of RCC-Shaw cells after cell viability assessment. After the Alamar Blue viability assay, RCC-Shaw tumor renal cells, seeded in 96-multi-well plates, were observed under an inverted microscope with 100× magnification, and brightfield images of the replicates were acquired. Here, a representative field of each condition is reported. For all the tested urine samples, a decrease in cell number was readily apparent compared to the control condition (CTR). Moreover, the polygonal tumor renal cells of the CTR became round-shaped, with the appearance of spikes (03FD, 09FL, and 13FP), extroflections (14FS, 15MS), and debris (12MP, 17MT), suggesting the occurrence of cell death processes. In each graph, data are reported as the percentage of the mean and the standard error of the mean (SEM) compared to the respective control conditions (non-exposed cells) assessed through the Mann–Whitney test, XLSTAT (*n* = 9–12; ** *p* < 0.01, **** *p* < 0.0001). Scale bar: 100 µm.

The observed cytotoxic effects of urine solutions on RCC-Shaw cells were corroborated by optical microscopy observations. Figure 6 depicts the brightfield microscopy analysis of the treated and control tumor renal cells after 48 h of incubation with dromedary urine solutions. The samples exhibiting cytotoxic effects through the Alamar Blue assay demonstrated a significant reduction in cellular density when microscopically examined compared to the control condition. For all the tested urine samples, a decrease in cell number was readily apparent with 100× magnification compared to the control condition (CTR, in the upper left panel). Moreover, alterations in the RCC-Shaw morphology after exposure to dromedary urine were observed: the polygonal RCC-Shaw cells of the control condition (CTR) became round-shaped (as shown for 04MF, 10MM samples), with the appearance of spikes (as can be seen in 03FD, 09FL, and 13FP), extroflections (14FS, 15MS), and debris (12MP, 17MT), suggesting the occurrence of cell death processes after exposure to dromedary urine.

### 2.4. Dromedary Urine Extracts Induce Apoptosis in Tumor Renal Cells

**Figure 7:**
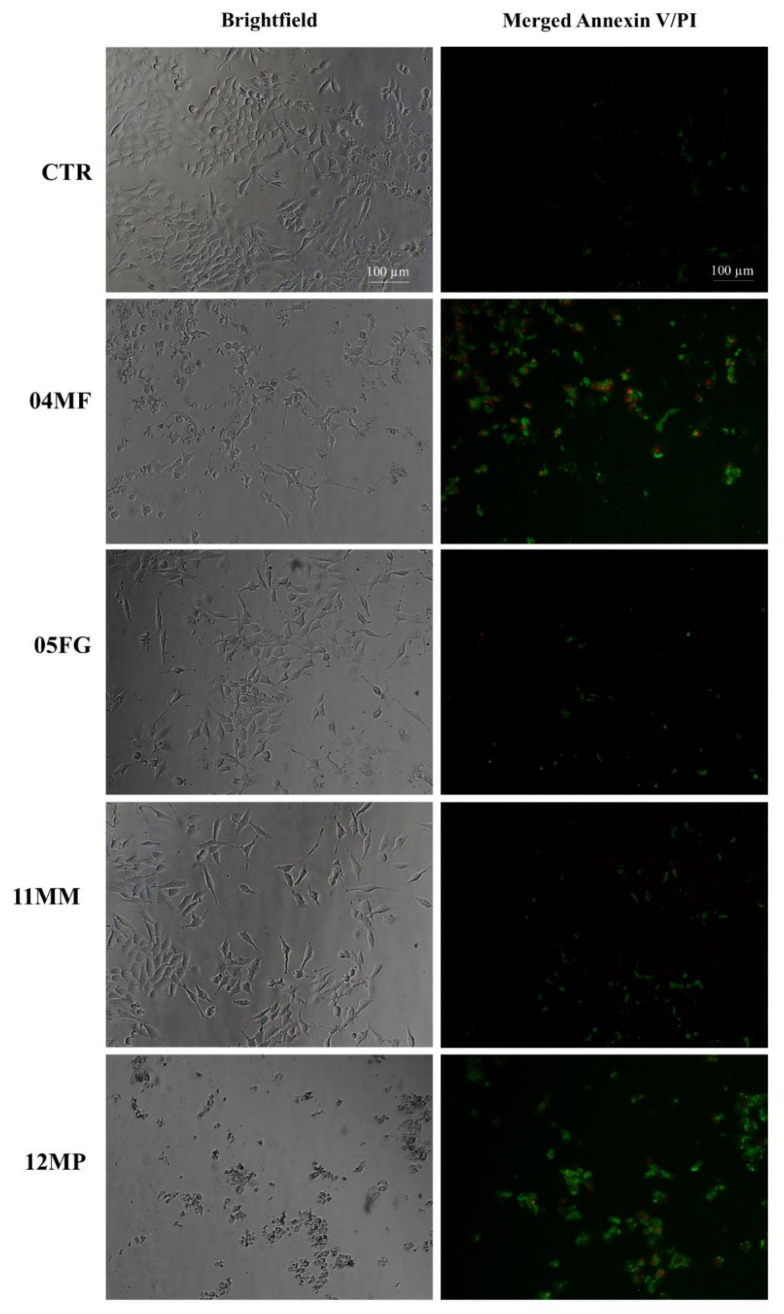
Annexin V-FITC/Propidium iodide double staining on RCC-Shaw cells after 24 h of treatment with dromedary urine solutions. Representative pictures of the brightfield images (on the left) and their respective green (Annexin V-FITC) and red (PI) fluorescence fields from the control condition (CTR), for cells exposed to urine from 04MF and 12MP dromedaries, and for cells exposed to 05FG and 11MM (on the right). Cells in the control (CTR) panels display negative results for both Annexin V-FITC and PI staining, and similar results can be observed for cells exposed to urine from 05FG and 1MM dromedaries. After exposure to urine solutions from 04MF and 12MP dromedaries for 24 h, cells exhibited strong green and red fluorescence, suggesting that apoptosis and necrosis are induced in RCC-Shaw cells. Scale bar: 100 µm.

In order to further investigate the molecular pathway underlying the observed death of RCC-Shaw tumor renal cells after exposure to dromedary urine, Annexin V-FITC/Propidium iodide (PI) double staining was performed. Briefly, RCC-Shaw cells were seeded in black 96 multi-well plates and exposed to dromedary urine solutions. After 24 h of treatment, cells underwent the Annexin V-FITC/PI assay and were analyzed through fluorescence microscopy. Figure 7 reports representative brightfield images and their respective green (Annexin V-FITC) and red (PI) fluorescence fields from the control condition, as well as from cells exposed to urine from 04MF and 12MP dromedaries, which exhibited a strong cytotoxic effect on RCC-Shaw cells according to the Alamar Blue assay, and from 05FG and 11MM, the latter of which only slightly affected cell viability. As shown in Figure 7, cells in the control (CTR) panels displayed negative results for both Annexin V-FITC and PI staining, indicating almost no apoptosis or necrosis, and similar results could be observed for cells exposed to urine from 05FG and 11MM dromedaries, which showed a weak Annexin V stain. However, after exposure to urine solutions from 04MF and 12MP animals for 24 h, cells exhibited strong green and red fluorescence, suggesting that apoptosis and necrosis are induced in RCC-Shaw cells. Therefore, the results from the Annexin V-FITC and PI double staining are in line with the cell viability outcomes. Cells in the control (CTR) panels display negative results for both Annexin V-FITC and PI staining, indicating almost no apoptosis or necrosis, and similar results can be observed for cells exposed to urine from 05FG and 1MM dromedaries (Figure 7). After exposure to urine solutions from 04MF and 12MP dromedaries for 24 h, cells exhibited strong green and red fluorescence, suggesting that apoptosis and necrosis were induced in RCC-Shaw cells (Figure 7).

### 2.5. NMR Metabolic Characterization of Dromedary Urine Samples

The chemical composition of the urine samples under investigation was defined through the study of the corresponding 1D ^1^H NOESY spectra (see Table 2). Such identification was made possible by performing a comparison with reference compounds and homo- and hetero-nuclear 2D NMR experiments. Many organic acids were detected, including acetic, lactic, pyruvic, azelaic, valeric, malonic, citric, and formic acids. Furthermore, typical derivatives of benzoic acid were found, such as hippuric acid and 4-hydroxy hippuric acid (HHA). Allantoin, most likely derived from the metabolism of uric acid, was well represented in all the samples under investigation. Hypoxanthine derivatives derived from the metabolism of purine were identified. Creatinine was detected in all samples, most likely derived from the metabolism of proteins. A few amino acid derivatives were identified, including carnitine, glycine, phenylacetylglycine, and valine. Furthermore, trimethylamine-N-oxide (TMAO) was contained in the samples under investigation as a result of potential oxidized products of quaternary ammonium salts, such as choline, betaine, and carnitine.

**Figure 8:**
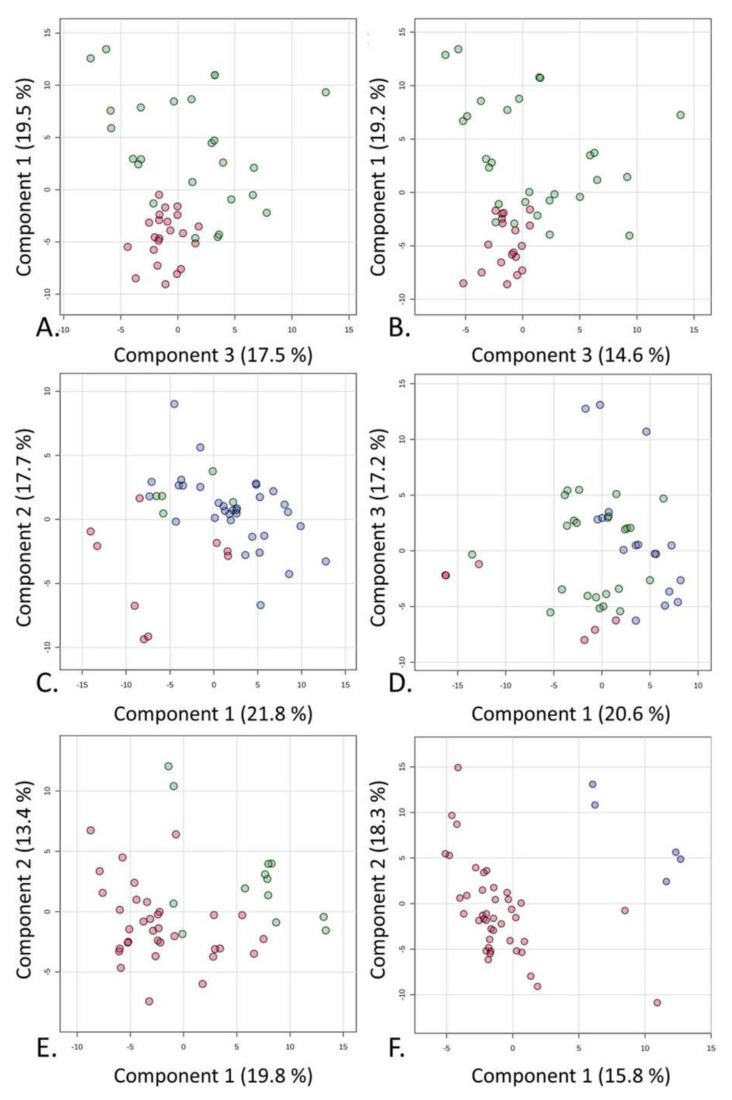
PLS-DA applied to the 46 spectra using UV-scaled 0.04 ppm-sized bucketing. The urine samples were assigned to the class of active (*p* < 0.0001), mildly active (0.0001 < *p* < 0.01), and inactive (*p* > 0.01) samples based on the calculated *p* value following the Mann–Whitney test. Score plots are shown between the selected components where the observations are indicated according to in vitro activity: “●” active, “●” mildly active, and “●” inactive. (**A**) HK-2 at 24 h. (**B**) HK-2 at 48 h. (**C**) Caki-1 at 24 h. (**D**) Caki-1 at 48 h. (**E**) RCC-Shawn at 24 h. (**F**) RCC-Shawn at 48 h.

Once the pool of the main metabolites was defined in the urine samples, a multivariate statistical analysis was attempted to determine the main variations in the metabolic compositions correlated with the observed bioactivity in the in vitro tests. As mentioned above, the urine samples were assigned to classes of active, mildly active, and inactive samples based on the calculated *p* value following the Mann–Whitney test. Specifically, the samples were considered active when *p* < 0.0001, mildly active when 0.0001 < *p* < 0.01, and inactive when *p* > 0.01. The spectroscopic data collected from the NMR measurements of the urine samples were subjected to a statistical investigation to unveil specific differences in the metabolic composition within the group of bioactive samples (red scores in Figure 8) vs. the mildly active (blue scores in Figure 8), and inactive samples (green scores in Figure 8). Three separate supervised analyses (PLS-DA) were conducted on the same spectroscopic data, and the bioactivity of different urine samples towards the three cell lines was considered to indicate classes of interest, both at 24 h and 48 h, i.e., for HK-2 (Figure 8A,B), Caki-1 (Figure 8C,D), and RCC-Shawn cells (Figure 8E,F). The models were validated through cross-validation to exclude any overfitting, and the coefficients were computed considering the contribution of five components. In all cases under investigation, the samples were grouped according to the class belonging to Component 1. In the specific case of the treatment of Caki-1 cells with the urine solutions, those samples that were defined as mildly active based on the in vitro experiments clustered closer to the inactive samples than the active ones, regardless of the duration of the treatment (Figure 8C,D).

**Figure 9:**
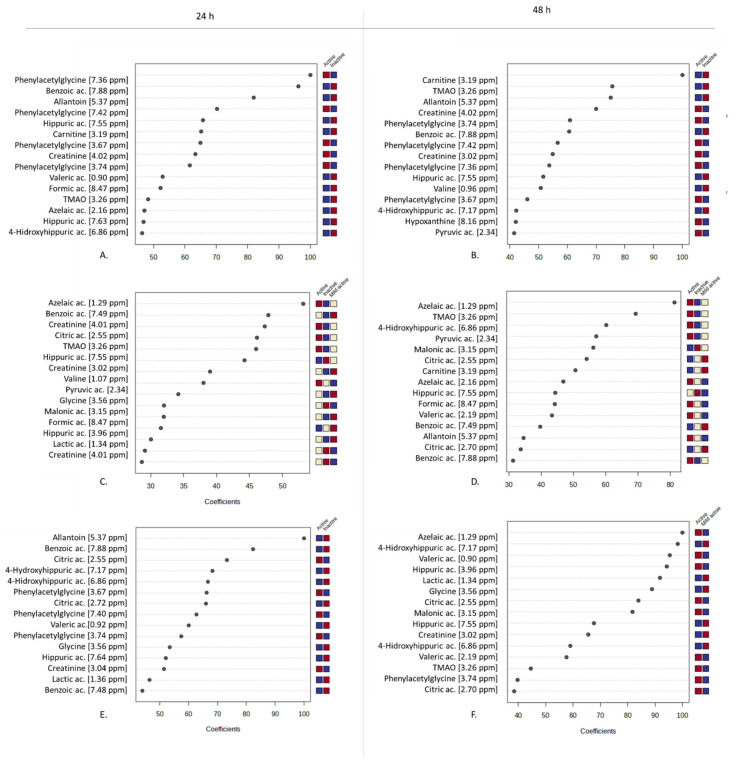
Analysis of the Variable Importance in Projection (VIP) identified by PLS-DA applied to the 46 spectra using UV-scaled 0.04 ppm-sized bucketing. The urine samples were assigned to classes of active (*p* < 0.0001), mildly active (0.0001 < *p* < 0.01), and inactive (*p* > 0.01) samples based on the calculated *p* value following the Mann–Whitney test. The colored boxes on the right indicate the relative concentrations of the corresponding metabolite in each group under study (■ high, ■ intermediate, and ■ low). (**A**). HK-2 at 24 h. (**B**). HK-2 at 48 h. (**C**). Caki-1 at 24 h. (**D**). Caki-1 at 48 h. (**E**). RCC-Shawn at 24 h. (**F**). RCC-Shawn at 48 h.

The analysis of the Variable Importance in Projection (VIP) was carried out to identify the pool of metabolites that more heavily changed from one class to the other following the PLS-DA performed and considering the treatment with urine solutions of the three cell lines at the two time points, i.e., 24 h and 48 h (Figure 9A–F).

The urine solutions that were active for HK-2 showed a relatively higher content of both phenylacetylglycine and creatinine, both at 24 h and 48 h of treatment. In addition, at this latter time point, a relatively higher content of hypoxanthine and pyruvic acid was also observed (Figure 9A,B). On the other side, azelaic acid and TMAO were contained in relatively higher amounts in the urine solutions that were active on Caki-1 cells, regardless of the treatment duration. In the case of RCC Shawn cells, the pool of metabolites contained in the urine solutions that were active at 24 h was more similar to that found for the urine solutions active towards HK-2 cells, with higher contents of phenylacetylglycine and creatinine. Conversely, after a longer treatment time (48 h), the following metabolites were found to be more abundant: organic acids (azelaic, valeric, hippuric, citric, and malonic acid), phenylacetylglycine, and TMAO.

## 3. Discussion

Natural matrices have historically been a valuable driver for drug discovery, even though natural product-based research is associated with some intrinsic issues related to the matrices’ properties and to the assays employed for the bioactivity assessment [14,27]. The present study aimed to evaluate the antiproliferative effects of dromedary urine, optimizing the experimental conditions to highlight their real impact on human renal cell lines and disentangling the contribution of hyperosmolarity to cytotoxicity [10,13]. Indeed, the tested dromedary urine samples showed a wide range of osmolarity values, all of which exceeded the physiological threshold of 290 mOsm/L. These observations suggest differences in urine concentration, possibly related to the interindividual variability of the sampled animals (sex, age, physiological status, and different levels of water consumption). In this context, the hyperosmolarity of urine samples should be considered a critical factor itself, which can alter bioactivity assay results due to its potential cytotoxicity, thus affecting the viability of cells not used to the hyperosmolar environment, such as proximal tubular epithelial cells [10].

Special focus was given to the role of fetal bovine serum (FBS), which is commonly employed as a supplement in animal cell cultures due to its essential nutrient content, supporting cell growth and proliferation. However, it is a complex mixture (and a natural matrix itself), and the elements within FBS remain partially unidentified. Their effects on cultured cells are poorly understood, even though they can interfere with in vitro experiments and affect the quality, reproducibility, and reliability of the studies. Indeed, FBS content may affect various cellular processes, including cell proliferation, differentiation, the modulation of molecular and cellular mechanisms, cell viability, and cytokine production [16,17,28,29]. Moreover, during in vitro experiments, the proteins and lipids contained in FBS can interact through reversible chemical bonds with the tested small molecules or drugs, possibly altering their outcomes and concealing the impacts of the tested chemicals on cellular processes [19,20]. Considering these observations, the experiments were carried out under serum-reduced conditions in order to minimize possible interfering factors derived from FBS. First, it was verified that the growth of the investigated cellular models did not suffer from 1% FBS supplementation. Subsequently, the hyperosmolarity tolerance threshold of HK-2, Caki-1, and RCC-Shaw cell lines under this culture condition was established. In a 1% FBS-supplemented cell culture medium, the hyperosmolarity tolerance threshold of 450 mOsm/L was identified for all the considered cell lines. A higher value of the hyperosmolarity tolerance threshold, i.e., 500 mOsm/L, was reported for HK-2 and Caki-1 cell lines in the presence of 10% FBS [10]. The lower amount of serum adopted in the present study could be responsible for the different outcomes, as a higher FBS content (10%) is known to affect in vitro stress responses [16,17,19], including responses in complex mixtures such as dromedary urine. Indeed, when diluted in 1% FBS-supplemented cell culture medium and administered to human renal cells, the urine showed a higher overall toxicity compared to the antiproliferative effect already reported on human renal cells [10], possibly due to the higher availability of small molecules in the serum-reduced condition [17,19,20]. In particular, the number of urine samples significantly affecting the viability of non-tumor HK-2 cells was lower than the samples that resulted in bioactivity towards the two tumor renal cell lines. Such evidence is in line with previous studies showing the different behavior of non-tumor vs. tumor cell lines [10,30,31,32]. The differential effects of urine samples on the tested cell lines may be attributed to the metabolite composition of the investigated urine. Each compound, whether alone or in combination with other molecules in the sample, could selectively affect cancer cells, which depend more strongly on specific metabolic pathways, such as those involved in energy production. As a result, they are more vulnerable to metabolic disruptions. In contrast, non-cancerous cells have greater metabolic flexibility and can adapt to certain metabolic changes, making them more resistant to specific alterations [8,33,34,35,36]. Interestingly, Caki-1 and RCC-Shaw exhibited opposite responses to dromedary urine exposure. Indeed, the Caki-1 cell line, derived from skin metastasis, initially showed a weak but statistically significant decline in viability for almost all the tested samples, but after 48 h, 6 of the 16 bioactive samples lost their effect on tumor renal cells, which appeared to adapt to the urine chemical composition and proliferate, in line with the ability of tumor cells to survive in challenging conditions [35,37]. On the other hand, all the tested dromedary urine samples were able to induce a strong and time-dependent decline in viability in the primary tumor-derived RCC-Shaw cells by roughly 85% of the cell toxicity caused by 04MF, 10MM, and 12MP samples, all derived from male animals, after 48 h of exposure. As demonstrated by the Annexin/PI staining profile of the tumor renal cells, the observed toxicity was mediated by apoptosis activation.

The 1D ^1^H NOESY analysis of urine samples revealed the presence of various organic acids, including pyruvic, acetic, lactic, malonic, formic, citric, hippuric, benzoic acids, valeric, azelaic, and 4-hydroxyhippuric acids. Also, valine, glycine, phenylacetylglycine, trimethylamine N-oxide (TMAO), carnitine, creatinine, allantoin, glycine, and hypoxanthine were found in the urine solutions. Following the chemometric analysis, relatively higher amounts of azelaic acid were found in urine samples that exhibited bioactivity against Caki-1 and RCC-Shaw cells but not against HK-2 cells. This observation can be explained by the relationship between azelaic acid and the NF-kB pathway. Azelaic acid (AA) is a naturally occurring nine-carbon dicarboxylic acid known for its antimicrobial and anticancer properties, particularly against various melanoma [38,39] and leukemia [40,41] cell lines. Additionally, it has anti-inflammatory [38] and radical oxygen species (ROS) scavenging activities [42]. Recent studies suggest that azelaic acid exerts its anti-inflammatory effect through binding to cyclooxygenase enzymes and its anticancer activity by targeting pirin, an iron-containing nuclear protein and transcription cofactor highly expressed in tumor cells [38,43], which is involved in the regulation of the NF-kB pathway [44]. A common gene mutation in clear cell renal carcinoma is the loss of function of the von Hippel-Lindau (VHL) gene, which is associated with NF-kB overexpression [45]. The inhibitory effect of azelaic acid on the NF-kB pathway might hinder the survival of RCC-Shaw and Caki-1 cells, thus explaining the observed results. In this context, the lower impact of dromedary urine on Caki-1 cells could be attributed to the fact that this cell line expresses a wild-type VHL gene [46,47,48], making it less affected by the activity of azelaic acid. Future research could explore AA efficacy as an adjuvant therapy in oncology, particularly in combination with existing treatments to enhance cancer cell sensitivity or reduce inflammatory side effects.

Among the other mentioned molecules highlighted by chemometric analysis, it was observed that relatively higher amounts of phenylacetyl glycine (PAGly) in urine samples might be responsible for the cytotoxicity against RCC-Shaw cells, but not against Caki-1 cells, despite affecting HK-2 renal cells. Phenylacetyl glycine (PAGly) is a phenylalanine derivative primarily synthesized by the gut microbiota [49,50]. Recently, PAGly has been proposed to target β2-adrenergic receptors (β2-AR), offering potential benefits for cerebral ischemia/reperfusion injury through the inhibition of receptor signaling [50]. Although the physiological role of β2-adrenergic receptors in the kidney remains underexplored, their protein and RNA expressions have been reported in various kidney subunits, including proximal tubules, glomeruli, and podocytes [51]. Moreover, blocking β2-adrenergic receptors has shown therapeutic benefits in clear cell renal cell carcinoma (ccRCC) with the VHL gene’s loss of function by reducing inflammation and oxidative stress [52,53], thus possibly explaining the observed effects on RCC-Shaw and Caki-1 cell lines. However, β2-AR antagonism has also been reported to increase renal production of proinflammatory cytokines, possibly hampering HK-2 proximal tubular cells [54,55]. Future studies should investigate the precise mechanism of action of PAGly in renal cancer models and assess its potential as a metabolic biomarker for therapeutic response prediction.

Nonetheless, it should be noted that the studied cellular models were exposed to a complex mixture of compounds, each contributing to the overall effect through either synergistic or antagonistic interactions, thereby complicating the interpretation of the results. In this regard, in the broader context of natural product research, the inherent variability in the composition of the tested natural matrices, such as urine in our assays, presents a significant challenge in ensuring the reproducibility of results. Factors such as individual metabolic differences, dietary habits, and the health status of animals can lead to fluctuations in metabolite profiles, potentially affecting the consistency of observed biological effects.

## 4. Materials and Methods

### 4.1. Urine Collection and Processing for In Vitro Assays

All the animals included in this study were reared on the same farm located in the Doñana National Park (southwestern Spain) and fed on the same diet, consisting of alfalfa, beet pulp, calcium carbonate, salt, and selenium. Urine sampling was conducted as previously described [10,56]. Briefly, for each animal, a sterile, single-use plastic bag was placed in a collection cone holder to recover mid-stream, first-morning-void urine during natural urination. Samples were collected from 17 dromedary camels (as detailed in Table 1, Section 2.2.) on a single day in November 2022. Immediately after collection, urine was transferred to 15 mL tubes and centrifuged at 2500× *g* for 5 min at 4 °C. The supernatant was filtered using a 0.22 μm PES syringe filter (Primo^®^, Euroclone S.p.A., Milan, Italy) and aliquoted. All aliquots were frozen at −20 °C and subsequently freeze-dried overnight at −50 °C in a vacuum below 0.5 mbar using a 5-pascal freeze drier (Speedvac SPD 140, Thermo Fisher Scientific, Milan, Italy). Lyophilized urine samples were reformed in ultrapure water at their initial volume immediately before the in vitro bioactivity experiments were conducted.

### 4.2. Assessment of Osmolarity in Dromedary Urine Samples

The osmolarity of dromedary urine samples was measured by a VAPRO^®^ vapor pressure osmometer 5600 (Wescor, Inc., Logan, UT, USA) with fresh 290 mmol/kg, 1000 mmol/kg, and 100 mmol/kg standard solutions, following the manufacturer’s instructions. Briefly, a single Whatman filter paper disc (Wescor, ss-033) was placed in the central depression of the holder using metal forceps, and 10 μL of the samples, diluted 1:2 with ultrapure water, was applied onto the disc. Saturated discs were promptly transferred to the vapor pressure osmometer sample holder, and osmolarity was determined. All samples were assessed three times, and osmolarity was calculated as the mean of the three determinations.

### 4.3. Cell Cultures

The human proximal tubule epithelial cell line (HK2) [57] and the human renal carcinoma cell line (Caki-1) [58], which serve as a model system of proximal tubule epithelium, were purchased from the American Type Culture Collection (ATCC, Manassas, VA, USA). RCC-Shaw cells are primary renal carcinoma cell lines derived from primary kidney tissue explants [59]. All three cell lines were cultured in Dulbecco’s modified Eagle’s medium with high glucose levels (Elabscience Bionovation Inc., Houston, TX, USA, EP-CM-L0032), supplemented with 10% fetal bovine serum (Euroclone S.p.A., Milan, Italy), in 25 cm^2^ culture flasks, and maintained at 37 °C and 5% CO_2_. They were regularly passaged upon reaching 80% confluency using a 0.25% trypsin-EDTA solution (Elabscience Bionovation Inc., USA, EP-CM-L0446).

### 4.4. Preparation of Hyperosmolar Solutions

Different osmolarity conditions of the cell culture medium increased and adjusted with mannitol, were assessed to determine the osmotic range at which no significant decrease in cell viability occurred. D-(-)-mannitol (Honeywell-Riedel-de Haën, Charlotte, North Carolina, USA, cat. no. 33440) was used as a reference osmolyte to investigate the tolerance limits of cultured cells to osmotic stress in serum-reduced conditions. Hyperosmolar solutions were prepared by adding the appropriate amount of D-(-)-mannitol powder to the culture medium supplemented with 1% FBS (whose osmolarity was 290 mOsm/L) to achieve osmolarities of 400, 450, 500, 600, and 700 mOsm/L, respectively. Once the D-(-)-mannitol was dissolved, the solutions were sterilized using 0.22 μm PES syringe filters (Primo^®^, Euroclone S.p.A., Milan, Italy).

### 4.5. Preparation of Urine Samples for In Vitro Bioactivity Test

All the freeze-dried urine samples were initially reconstructed in ultrapure water to restore their original volume. Subsequently, they were properly diluted using the 1% FBS-supplemented cell culture medium, which is referred to as a serum-reduced condition as it remained below the 450 mOsm/L threshold (as outlined in Results, Section 3). The obtained urine solutions were sterilized using 0.22 μm PES syringe filters (Primo^®^, Euroclone S.p.A., Milan, Italy).

### 4.6. Cell Viability Assay

HK-2, Caki-1, and RCC-Shaw cells were seeded in 96-well plates at a density of 15,000 cells per well in the growth medium, supplemented with 10% FBS and allowed to adhere overnight. Subsequently, the cells were treated with hyperosmolar media adjusted with D-(-)-mannitol or with urine solutions, both prepared in a 1% FBS-supplemented medium for 24 and 48 h at 37° C and 5% CO_2_. Cell viability was assessed under control conditions (cells exposed to the 1% FBS-supplemented medium) and experimental conditions using the Alamar Blue assay (Immunological Sciences, Rome, Italy), according to the manufacturer’s instructions. This assay relies on the ability of living cells to convert the blue non-fluorescent dye (resazurin) to a red fluorescent dye (resorufin) via mitochondrial reductase. Following a 45 min incubation with resazurin, the fluorescence of resorufin was measured at λ_Ex/Em_ 535/590 nm using an FLUOstar^®^ Omega microplate reader (BMG LABTECH GmbH, Ortenberg, Germany). All the experiments were performed in triplicate at least three times.

### 4.7. Annexin V-FITC/Propidium Iodide Double Staining

The Annexin V-FITC/PI assay can differentiate viable cells (Annexin V− and PI−) from early apoptosis-staged cells (Annexin V+ and PI−) or from late apoptosis (Annexin V+ and PI+) and necrosis (Annexin V− and PI+) stages. The Annexin V-FITC/PI Apoptosis Kit (Elabscience Inc, Houston, TX, USA, E-CK-A211) was performed following the manufacturer’s instructions. Briefly, RCC-Shaw cells were seeded in black 96 multi-well plates (Greiner Bio-One S.r.l, Cassina de Pecchi, Italy, cod. 655090) at a density of 10,000 cells/well in the growth medium and allowed to become attached overnight. Afterward, cells were exposed to dromedary urine diluted in a serum-reduced cell culture medium for 24 h and were stained for Annexin V-FITC and PI for 15 min at room temperature. Subsequently, the plates were placed in a fluorescence microscope (TE2000 inverted microscope by Nikon, Turin, Italy) equipped with a Nikon Digital SIGHT camera and the NIS Elements software (version 3.00 SP7; Nikon, Turin, Italy) to capture fluorescence images, with excitation/emission wavelengths of 485/520 nm for Annexin V-FITC and 535/615 nm for PI with 100× magnification.

### 4.8. Nuclear Magnetic Resonance (NMR) Analysis

Freeze-dried urine samples (150 mg) were reconstituted in 2.0 mL phosphate buffer (100 mM, pH 7.4) containing 0.1 mM TSP-d4. Next, the samples were vortexed at 2500 rpm for 2 min (Advanced Vortex Mixer ZX3, VELP Scientifica Srl, Usmate, Italy). Next, three aliquots of 600 μL each were obtained and transferred into three different NMR tubes to achieve three analytical replicates. The 1D 1H NOESY spectra were recorded using a Bruker Avance I 400 MHz spectrometer equipped with an autosampler and a 5 mm inverse probe (Bruker BioSpin GmbH, Ettlingen, Germany). The 1D 1H NOESY spectra were acquired using the pulse program (noesygppr1d). The following parameters were applied: number of scans: 128; data points: 64 K; spectral width: 8013 Hz; 90° pulse angle: 8.16 μs; acquisition time: 4.09 s; mixing time: 10 ms; and recycle delay: 6 s. Each spectrum was acquired using the Topspin 2.1 software (Bruker BioSpin GmbH) under an automatic process that lasted around 22 min and included sample loading, temperature stabilization for 5 min at 298.2 K, tuning, matching, and shimming. The NMR raw data (Free Induction Decays, FIDs) were processed using the software MestReNova 11.0 (Mestrelab Research SL, Santiago de Compostela, Spain). The FIDs were zero-filled to a 128 K number of points and then underwent Fourier transformation through the application of an exponential multiplication function with a line broadening of 0.1 Hz. The phase and baseline were automatically corrected, and the TSP-d4 singlet signal set at δ = 0.00 ppm was used as a chemical shift reference.

### 4.9. Chemometric Analysis

The raw data (FIDs) derived from the 1D 1H NOESY measurements were processed using Mestrelab and segmented into regular-sized (0.04 ppm) intervals (buckets) in the range of [10.50, 0.50] ppm. The underlying area of each bucket was calculated and normalized to the total intensity. The areas of the buckets in the region [5.13, 4.69] ppm, corresponding to the residual water signal, were set to 0. The data were stored in a table with one sample per row and one variable (bucket) per column. The data matrices were imported into MetaboAnalyst 5.0, and buckets were subjected to mean-centering and divided by the standard deviation of each variable (Unit Variance scaling). PLS-DA was used as a supervised method that uses multivariate regression techniques to extract information via a linear combination of original variables (X) that could predict the class membership (Y). The PLS regression was performed using the plsr function provided by the R package version 2.1-0 [60]. The classification and cross-validation were performed using the corresponding wrapper function offered by the caret package [61]. The caret package is available online: https://topepo.github.io/caret/ (accessed on 24 November 2024). To assess the significance of class discrimination, a permutation test was performed. In each permutation, a PLS-DA model was built between the data (X) and the permuted class labels (Y) using the optimal number of components determined by cross-validation for the model based on the original class assignment [62]. To estimate the predictive ability of the model, the performance measure Q2 was calculated via cross-validation (CV). In each CV, the predicted data were compared with the original data, and the sum of squared errors was calculated. The prediction error was then summed over all the samples (the Predicted Residual Sum of Squares or PRESS). The variable importance was measured in PLS-DA using the Variable Importance in Projection (VIP), which is a weighted sum of squares of the PLS loadings, taking into account the amount of explained Y-variation in each dimension.

### 4.10. Statistical Analysis

Data are presented as the means ± standard errors (SEM). The effect of the treatments, i.e., on (i) hyperosmotic media and (ii) urine solutions, was analyzed with XLSTAT software (version 2023.3.0.1415) using the Mann–Whitney test. Differences were considered statistically significant at *p* < 0.05. In particular, statistical significance was assessed compared to the control condition and indicated as follows: * *p* < 0.05, ** *p* < 0.01, *** *p* < 0.001, **** *p* < 0.0001. The number of observations (*n*) for each condition is indicated in the figure legends.

## 5. Conclusions

This study underscores the critical importance of determining optimal experimental conditions when evaluating the potential bioactivity of natural matrices in vitro, particularly dromedary urine, which presents specific challenges, such as osmolarity issues. The amount of fetal bovine serum, which can alter the sensitivity of the tested cell lines to the complex mixture of molecules present in urine, plays a pivotal role in the outcomes of cell-based assays, as demonstrated in our cytotoxicity evaluations. Furthermore, it is crucial to correlate in vitro evaluations with a thorough characterization of the chemical composition of the tested matrices. Indeed, the intrinsic variability of urine, which limits the applications of this natural matrix within the field of natural product-related drug discovery, can be reduced by ensuring appropriate experimental conditions, which is essential for enabling more reliable statistical analyses when comparing metabolic profiles. In this context, metabolomics approaches play a pivotal role in highlighting the most promising compounds present in this natural matrix, such as azelaic acid and phenylacetyl glycine, allowing us to propose dromedary urine as a sustainable source of bioactive molecules with potential biomedical applications. Overall, our findings on AA and PAGly, obtained by metabolomics analysis, emphasize the importance of identifying bioactive molecules from natural matrices in order to shape new therapeutic strategies, particularly in the context of precision medicine, where individual metabolic profiles may guide treatment decisions and enhance drug efficacy.

Moreover, repurposing camel urine—a by-product often considered a waste material—as a source of bioactive molecules aligns with the principles of green chemistry and circular economy, offering an innovative avenue for the value-added utilization of natural resources.

## Figures and Tables

**Table 1:** Measurement of dromedary urine samples’ osmolarity (mOsm/L) through a vapor pressure osmometer.

Dromedary ID	Sex, Age (years)	Urine Osmolarity (mOsm/L)
**01MA**	Male, 15	1112
**02MC**	Male, 13	1648
**03FD**	Female, 36	1000
**04MF**	Male, 2.5	1725
**05FG**	Female, 12.5	541
**06FG**	Female, 6	784
**07FG**	Female, 5	691
**08FK**	Female, 1.5	1095
**09FL**	Female, 12	1653
**10MM**	Male, 30	1727
**11MM**	Male, 14	424
**12MP**	Male, 34	1417
**13FP**	Female, 5	1917
**14FS**	Female, 20	1465
**15MS**	Male, 31	1513
**16MS**	Male, 5	1976
**17MT**	Male, 21	1915

**Table 2:** List of the main metabolites detected in the aqueous extracts of the analyzed urine samples.

Metabolite	δ (ppm) and Multiplicity Details ^a^
Valine	0.96 (d, 7.0 Hz); 1.07 (d, 7.0 Hz)
Lactic ac.	1.34 (d, 6.9 Hz); 4.14 (q)
Acetic ac.	1.93 (s)
Pyruvic ac.	2.34 (s)
Azelaic ac.	1.29 (m); 1.54 (m); 2.16 (t, 3.2 Hz)
Valeric ac.	0.9 (t, overlap); 1.31 (m); 1.53 (m); 2.19 (t, overlap))
Creatinine	3.02 (s); 4.01 (s)
Malonic ac.	3.15 (s)
Carnitine	3.19 (s)
Trimethylamine *N*-oxide (TMAO)	3.26 (s)
Glycine	3.56 (s)
Allantoin	5.37 (s)
Citric ac.	2.55 (d, 15.8 Hz); 2.70 (d, 15.7 Hz)
Phenylacetyl-glycine	3.67 (s); 3.74 (s); 7.36 (m); 7.42 (m)
Hippuric acid	7.55 (m); 7.63 (m); 7.83 (m); 3.96 (s)
4-Hydroxyhippuric ac.	6.86 (d, 8.5 Hz); 7.17 (d, 8.5 Hz)
Benzoic acid	7.88 (m); 7.56 (m); 7.49 (m)
Hypoxanthine	8.14 (s); 8.16(s)
Formic ac.	8.47 (s)

^a^ The resonance frequencies of the signals are indicated as delta (δ), and the units are given in part per million (ppm); multiplicity patterns are described as follows: (s) singlet, (d) doublet, (t) triplet, (q) quartet, (m) multiplet, and (dd) doublet of doublets.

## Data Availability

The data presented in this study are available upon request from the corresponding author.

## Supplementary Materials

The following supporting information can be downloaded at: https://www.mdpi.com/article/10.3390/molecules30040821/s1, Table S1: Cell viability assay on non-tumor HK-2 cells after treatment with dromedary urine samples. Data are reported as the percentage of the mean and the standard error of the mean (SEM) com-pared to the respective control conditions (non-exposed cells) and samples were labeled as (i) inactive when *p* > 0.01, (ii) mild bioactive when 0.01 < *p* < 0.0001, (iii) bioactive when *p* < 0.0001 (Mann-Whitney Test, XLSTAT, n = 9–15). Table S2: Cell viability assay on tumor Caki-1 cells after treatment with dromedary urine samples. Data are reported as the percentage of the mean and the standard error of the mean (SEM) compared to the respective control conditions (non-exposed cells) and samples were labeled as (i) inactive when *p* > 0.01, (ii) mild bioactive when 0.01 < *p* < 0.0001, (iii) bioactive when *p* < 0.0001 (Mann-Whitney Test, XLSTAT, n = 9–18). Table S3: Cell viability assay on tumor RCC-Shaw cells after treatment with dromedary urine samples. Data are reported as the percentage of the mean and the standard error of the mean (SEM) compared to the respective control conditions (non-exposed cells) and samples were labeled as (i) inactive when *p* > 0.01, (ii) mild bioactive when 0.01 < *p* < 0.0001, (iii) bioactive when *p* < 0.0001 (Mann-Whitney Test, XLSTAT, n = 9–12).
