# Supplementary material for: Serum Starvation Enhances the Antitumor Activity of Natural Matrices: Insights into Bioactive Molecules from Dromedary Urine Extracts
Source: Molecules. 2025 Feb 10;30(4):821. doi: 10.3390/molecules30040821 (PMC11858132; doi:10.3390/molecules30040821)
Supplement: Supplementary file 1 [file molecules-30-00821-s001.zip › molecules-3428556-supplementary.pdf]

Serum Starvation Enhances the Antitumor Activity of Natural Matrices: Insights into Bioactive Molecules from Dromedary Urine Extracts

Maria Noemi Sgobba [1,*,†], Biagia Musio [2,†], Carlos Iglesias Pastrana [3], Stefano Todisco [2], Nikola Schlosserovà [1], Federica Mastropirro [1], Maria Favia [4], Antonio Radesco [5], Iola F. Duarte [6], Anna De Grassi [1], Mariateresa Volpicella [1], Vito Gallo [2,7], Ciro Leonardo Pierri [8,*], Elena Ciani [1] and Lorenzo Guerra [1]

[1]Department of Biosciences, Biotechnologies and Environment, University of Bari "Aldo Moro", Via Orabona, 4, 70125, Bari, Italy
[2]Department of Civil, Environmental, Land, Building Engineering and Chemistry (DICATECh), Polytechnic University of Bari, Via Orabona, 4, I-70125 Bari, Italy
[3]Faculty of Veterinary Sciences, Department of Genetics, University of Córdoba, 14071 Córdoba, Spain
[4]Department of Translational Biomedicine and Neurosciences (DiBraiN), University of Bari "Aldo Moro", Piazza Giulio Cesare, 70124, Bari, Italy
[5]Istituto Tumori "Giovanni Paolo II" I.R.C.C.S., Viale Orazio Flacco 65, 70124, Bari, Italy
[6]Department of Chemistry, CICECO – Aveiro Institute of Materials and LAQV-REQUIMTE, University of Aveiro, Aveiro, Portugal
[7]Innovative Solutions S.r.l.—Spin-Off Company of the Polytechnic University of Bari, Zona H 150/B, I-70015 Noci (BA), Italy
[8]Department of Pharmacy – Pharmaceutical Sciences, University of Bari Aldo Moro, Via Orabona, 4, 70125, Bari, Italy
* Correspondence: maria.sgobba@uniba.it; ciro.pierri@uniba.it

† These authors equally contributed.

Supplementary materials

Table S1. Cell viability assay on non-tumor HK-2 cells after treatment with dromedary urine samples. Data are reported as the percentage of the mean and the standard error of the mean (SEM) com-pared to the respective control conditions (non-exposed cells) and samples were labeled as (i) inactive when $p>0.01$, (ii) mild bioactive when $0.01<p<0.0001$, (iii) bioactive when $p<0.0001$ (Mann-Whitney Test, XLSTAT, n = 9-15).

Sample ID	24H			48H		
	**Mean ± SEM (%)**	***p*-value**	**Bioactivity**	**Mean ± SEM (%)**	***p*-value**	**Bioactivity**
**01MA**	97.68 ± 1.80	0.0930	Inactive	96.95 ± 0.82	0.0930	Inactive
**02MC**	106.86 ± 2.57	0.8501	Inactive	95.93 ± 2.31	0.1134	Inactive
**03FD**	89.71 ± 2.10	9.71E-06	**Bioactive**	87.13 ± 2.94	9.72E-06	**Bioactive**
**04MF**	85.11 ± 3.68	6.99E-06	**Bioactive**	84.75 ± 2.42	1.79E-06	**Bioactive**
**05FG**	102.84 ± 2.04	0.9819	Inactive	96.29 ± 2.41	0.2273	Inactive
**06FG**	97.70 ± 1.35	0.2426	Inactive	101.07 ± 2.12	0.9487	Inactive
**07FG**	105.29 ± 3.06	0.1014	Inactive	101.11 ± 4.52	0.6063	Inactive
**08FK**	90.66 ± 2.82	0.0273	Inactive	83.80 ± 4.58	0.0122	Inactive
**09FL**	95.77 ± 3.03	0.1935	Inactive	99.69 ± 3.94	0.9095	Inactive
**10MM**	85.05 ± 2.64	9.03E-08	**Bioactive**	76.36 ± 2.84	2.59E-07	**Bioactive**
**11MM**	102.79 ± 5.95	0.6729	Inactive	99.56 ± 1.92	0.6664	Inactive
**12MP**	82.37 ± 2.68	1.29E-08	**Bioactive**	75.24 ± 3.67	1.22E-06	**Bioactive**
**13FP**	85.99 ± 3.22	5.16E-08	**Bioactive**	88.79 ± 2.07	2.51E-06	**Bioactive**
**14FS**	89.98 ± 1.36	2.58E-08	**Bioactive**	86.43 ± 2.21	7.74E-07	**Bioactive**
**15MS**	86.50 ± 2.55	1.07E-07	**Bioactive**	82.97 ± 3.19	9.03E-08	**Bioactive**
**16MS**	91.87 ± 2.64	0.1614	Inactive	91.99 ± 1.70	0.0142	Inactive
**17MT**	90.33 ± 2.10	7.42E-06	**Bioactive**	91.28 ± 1.79	0.00011	**Mild**

Table S2. Cell viability assay on tumor Caki-1 cells after treatment with dromedary urine samples. Data are reported as the percentage of the mean and the standard error of the mean (SEM) compared to the respective control conditions (non-exposed cells) and samples were labeled as (i) inactive when p>0.01, (ii) mild bioactive when 0.01<p<0.0001, (iii) bioactive when p<0.0001 (Mann-Whitney Test, XLSTAT, n = 9-18).

**Sample ID**	**24H**			**48H**		
	**Mean ± SEM (%)**	***p*-value**	**Bioactivity**	**Mean ± SEM (%)**	***p*-value**	**Bioactivity**
**01MA**	92.80 ± 2.17	0.0086	**Mild**	101.28 ± 0.5773	0.2402	Inactive
**02MC**	78.28 ± 3.19	0.0018	**Mild**	88.76 ± 3.42	0.0314	Inactive
**03FD**	91.40 ± 2.90	0.0052	**Mild**	96.16 ± 2.02	0.0294	Inactive
**04MF**	90.42 ± 4.30	0.0008	**Mild**	86.27 ± 2.63	0.0004	**Mild**
**05FG**	89.57 ± 1.14	0.0002	**Mild**	87.06 ± 3.03	0.0021	**Mild**
**06FG**	90.36 ±1.43	0.0192	**Mild**	94.57 ± 1.49	0.0879	Inactive
**07FG**	92.09 ± 2.06	0.2169	Inactive	94.29 ± 2.74	0.1512	Inactive
**08FK**	78.44 ± 2.32	9.47E-07	**Bioactive**	76.62 ± 3.21	9.47E-07	**Bioactive**
**09FL**	85.50 ± 2.87	9.72E-06	**Bioactive**	90.30 ± 1.67	0.0058	**Mild**
**10MM**	84.41 ± 3.64	9.58E-05	**Bioactive**	85.47 ± 3.36	0.0005	**Mild**
**11MM**	86.77 ± 2.51	0.0315	Inactive	95.89 ± 1.91	0.2973	Inactive
**12MP**	91.41 ± 4.29	0.0064	**Mild**	78.35 ± 4.42	3.43E-05	**Bioactive**
**13FP**	92.48 ± 2.14	0.0027	**Mild**	96.26 ± 1.74	0.0742	Inactive
**14FS**	87.19 ± 3.45	0.0058	**Mild**	93.97 ± 1.92	0.0086	**Mild**
**15MS**	83.62 ± 3.64	0.0002	**Mild**	93.07 ± 1.81	0.0027	**Mild**
**16MS**	86.95 ±1.31	0.0005	**Mild**	94.64 ± 2.25	0.1614	Inactive
**17MT**	82.19 ± 3.26	0.0016	**Mild**	97.63 ± 1.55	0.1369	Inactive

Table S3. Cell viability assay on tumor RCC-Shaw cells after treatment with dromedary urine samples. Data are reported as the percentage of the mean and the standard error of the mean (SEM) compared to the respective control conditions (non-exposed cells) and samples were labeled as (i) inactive when p>0.01, (ii) mild bioactive when 0.01<p<0.0001, (iii) bioactive when p<0.0001 (Mann-Whitney Test, XLSTAT, n = 9-12).

**Sample ID**	**24H**			**48H**		
	**Mean ± SEM (%)**	***p*-value**	**Bioactivity**	**Mean ± SEM (%)**	***p*-value**	**Bioactivity**
**01MA**	70.29 ± 3.62	1.31E-06	**Bioactive**	27.76 ± 3.22	2.45E-06	**Bioactive**
**02MC**	69.88 ± 2.84	1.97E-06	**Bioactive**	28.34 ± 3.09	6.8E-06	**Bioactive**
**03FD**	67.41 ± 4.58	3.83E-06	**Bioactive**	56.18 ± 4.18	1.02E-06	**Bioactive**
**04MF**	34.57 ± 6.22	4.02E-06	**Bioactive**	14.51 ± 1.55	3.85E-08	**Bioactive**
**05FG**	94.82 ± 3.13	0.2238	Inactive	83.87 ± 4.17	0.0034	**Mild**
**06FG**	68.40 ± 4.89	2.94E-05	**Bioactive**	42.83 ± 3.39	2.45E-06	**Bioactive**
**07FG**	90.19 ± 3.28	0.048	Inactive	84.92 ± 3.70	0.0015	**Mild**
**08FK**	85.47 ± 3.15	0.0122	Inactive	60.28 ± 3.89	6.8E-06	**Bioactive**
**09FL**	85.09 ± 3.99	0.0105	Inactive	76.99 ± 4.16	7.4E-06	**Bioactive**
**10MM**	21.00 ± 2.29	4.02E-06	**Bioactive**	12.68 ± 1.04	2.37E-07	**Bioactive**
**11MM**	97.77 ± 1.77	0.5689	Inactive	82.25 ± 3.42	4.4E-05	**Bioactive**
**12MP**	42.57 ± 3.75	9.99E-08	**Bioactive**	12.69 ± 1.50	3.85E-08	**Bioactive**
**13FP**	58.56 ± 3.51	2.45E-06	**Bioactive**	30.05 ± 3.75	3.54E-09	**Bioactive**
**14FS**	57.85 ± 3.71	6.12E-07	**Bioactive**	24.36 ± 4.81	7.4E-07	**Bioactive**
**15MS**	48.29 ± 3.63	4.02E-06	**Bioactive**	26.18 ± 3.93	9.31E-09	**Bioactive**
**16MS**	56.84 ± 4.43	4.08E-06	**Bioactive**	34.67 ± 3.67	7.54E-09	**Bioactive**
**17MT**	67.87 ± 3.21	1.16E-06	**Bioactive**	15.47 ± 2.02	3.85E-08	**Bioactive**
